# A Body‐Temperature‐Triggered In Situ Softening Peripheral Nerve Electrode for Chronic Robust Neuromodulation

**DOI:** 10.1002/advs.202412361

**Published:** 2024-12-06

**Authors:** Xueyang Ren, Wenjie Tang, Yuehui Yuan, Shisheng Chen, Fangzhou Lu, Jinyang Mao, Jidan Fan, Xufeng Wei, Ming Chu, Benhui Hu

**Affiliations:** ^1^ School of Biomedical Engineering and Informatics Nanjing Medical University Nanjing 211166 China; ^2^ Jinan Microecological Biomedicine Shandong Laboratory Jinan 250000 China; ^3^ School of Electronic Science and Engineering Southeast University Nanjing 211189 China; ^4^ Department of Endocrinology The First Affiliated Hospital of Nanjing Medical University Nanjing 210029 China; ^5^ The Affiliated Taizhou People's Hospital of Nanjing Medical University Taizhou School of Clinical Medicine Nanjing Medical University Taizhou 225300 China; ^6^ Department of Cardiovascular Medicine The First Affiliated Hospital of Nanjing Medical University Nanjing 210029 China; ^7^ State Key Laboratory of Reproductive Medicine and Offspring Health Affiliated Stomatological Hospital Nanjing Medical University Nanjing 210029 China

**Keywords:** in situ softening, myocardial remodeling, neuromodulation, peripheral nerve electrodes

## Abstract

Implantable peripheral nerve electrodes are crucial for monitoring health and alleviating symptoms of chronic diseases. Advanced compliant electrodes have been developed because of their biomechanical compatibility. However, these mechanically tissue‐like electrodes suffer from unmanageable operating forces, leading to high risks of nerve injury and fragile electrode‐tissue interfaces. Here, a peripheral nerve electrode is developed that simultaneously fulfills the criteria of body temperature softening and tissue‐like modulus (less than 0.8 MPa at 37 °C) after implantation. The central core is altered from the tri‐arm crosslinker to the star‐branched monomer to kill two birds (close the translation temperature to 37 °C and decrease the modulus after implantation) with one stone. Furthermore, the decreased interfacial impedance (325.1 ± 46.9 Ω at 1 kHz) and increased charge storage capacity (111.2 ± 5.8 mC cm^−2^) are achieved by an in situ electrografted conductive polymer on the strain‐insensitive conductive network of Au nanotubes. The electrodes are readily wrapped around nerves and applied for long‐term stimulation in vivo with minimal inflammation. Neuromodulation experiments demonstrate their potential clinical utility, including vagus nerve stimulation in rats to suppress seizures and alleviation of cardiac remodeling in a canine model of myocardial infarction.

## Graphical abstract

This study develops a peripheral nerve electrode, which achieves body temperature softening and a tissue‐like modulus (< 0.8 MPa) through the central core transition from a tri‐arm crosslinker to a star‐branched monomer, along with enhanced charge capacity via in situ electrografted conductive polymers. The electrode minimizes nerve injury, ensures long‐term stability, and demonstrates potential in neuromodulation for seizure suppression and alleviation of cardiac remodeling.

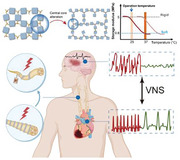

## 1 Introduction

**Figure 1:**
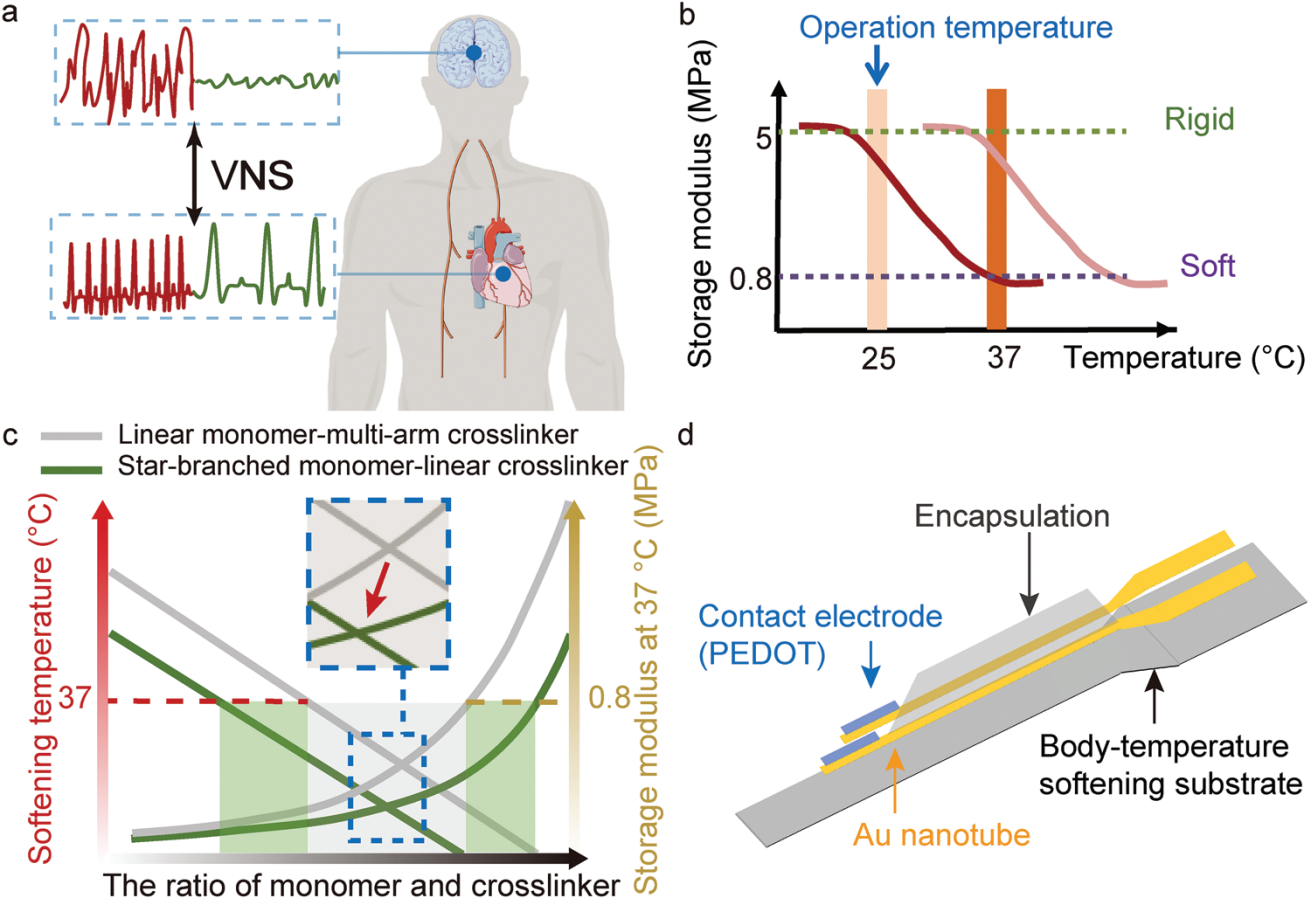
The concept of BIS‐PNE. a) Schematic diagram of the conceptual VNS neuromodulation for reducing heart rate and inhibiting abnormal neuron discharge. b) Diagram of in situ softening based on dynamic mechanical analysis. c) The schematic diagram of change curves of the temperature of soften and storage modulus at body temperature with the increase of the ratio of monomer and crosslinker. d) The layout of the BIS‐PNE.

Long‐term intermittent peripheral nerve electrical stimulation has emerged as a novel therapeutic strategy for chronic diseases, such as cardiovascular diseases and refractory epilepsy^[^ ^1^ ^]^ (vagus nerve stimulation suppressing abnormal neuronal activity, **Figure** **1**a). Compared to non‐invasive strategies, implantable peripheral nerve electrodes directly contact target nerve bundles, allowing precise perception and modulation of neural activity. Given the slippery nature of epineurium in biofluids,^[^ ^2^ ^]^ a robust electrode‐nerve interface is essential to resist interference from changes in interfacial impedance during body motion.^[^ ^3^, ^4^, ^5^, ^6^ ^]^ Although mechanical locking and self‐adhesive electrodes offer effective strategies to enhance interface robustness,^[^ ^7^, ^8^ ^]^ irreversible nerve damage (from mechanical constraints) and interface failure (from apoptotic epineural cell contamination) are inevitable.^[^ ^9^, ^10^, ^11^ ^]^ Recent work on mechanically tissue‐like electrodes has achieved good biocompatibility and motion adaptability by eliminating mechanical mismatches.^[^ ^12^, ^13^ ^]^ However, the modulus of these ultra‐soft electrodes is below the human perception threshold, hindering proprioceptive feedback to surgeons. The lack of feedback makes it difficult for surgeons to manage the operating force, resulting in unconformable contact between the electrode and nerves (excessive stretching generating residual stress or excessive bending creating length edges), and even directly leading to electrode failure.

Temporarily hardening of the electrodes during implantation could provide adequate mechanical feedback to the surgeons.^[^ ^14^ ^]^ The conventional approaches include employing retractable tools or coating electrodes with hard and bioresorbable polymers.^[^ ^15^, ^16^ ^]^ Unfortunately, the rigid and retractable tool elevates the risk of implantation‐induced injury, while the degradation products alter the tissue microenvironment, increasing the risk of metabolic abnormalities.^[^ ^17^ ^]^ Recent advancements in materials science have facilitated the development of electrode substrates that can soften under intracorporeal conditions without disrupting the local physiological environment. These advancements can be categorized into hydrogel swelling^[^ ^18^ ^]^ and silk supercontraction^[^ ^19^ ^]^ upon wetting or shape memory polymers (SMPs)^[^ ^20^ ^]^ responding to body temperature. However, the significant expansion of the hydrogel substrate (≈60–70%) results in structural changes of the electrode and generation of leakage current over time. The silk‐based substrates are unable to tolerate enzymatic degradation in vivo. Thus, both hydrogel and silk are not applicable for chronic implantation. Alternatively, the storage modulus of SMP‐based substrates prepared from hydrophobic polymers remains greater than 10 MPa after softening,^[^ ^21^ ^]^ which is much higher than that of nerve tissue (0.6 MPa).^[^ ^22^ ^]^ Although introducing hydrophilic molecular chains can reduce the modulus of the substrate to 0.3 MPa by absorbing body fluids, this approach inevitably leads to current leakage.^[^ ^23^ ^]^ To date, the development of peripheral nerve electrodes that can both soften under physiological conditions and achieve a resultant tissue‐like modulus remains an unmet challenge.

The SMP‐based substrate, polymerized by hydrophobic linear monomers and multi‐arm crosslinkers, cannot meet the requirements of body temperature (37 °C) softening and low modulus after softening (0.8 MPa), as illustrated by the dynamic mechanical analysis (DMA)^[^ ^24^ ^]^ (Figure 1b, dotted line). We hypothesize that using a star‐branched polymer/linear crosslinker, instead of a linear polymer/multi‐arm crosslinker, would cause a downward shift in the cross‐point of the curves depicting the variation of softening temperature and storage modulus at different crosslinker contents (as shown in Figure 1c, enlarged image). This would broaden the range of monomer and crosslinker ratios that meet the above requirements. Two arguments support this hypothesis. First, the mobility of each arm of the star‐branched monomer, which has a shorter arm length than the linear monomer (same molecular weight for these two monomers), is more restricted by the central core (central connect point of the star‐branched polymer). As a result, chain folding and regular packing of monomers decrease, which leads to a reduction of crystallinity and melting temperature. It is beneficial for lowering the softening temperature to below the body temperature.^[^ ^25^ ^]^ Second, the linear crosslinker is introduced to create crystal defects and prohibit the rapid increase of crosslink density, resulting in a lower modulus after softening.^[^ ^26^ ^]^ Therefore, an electrode substrate polymerized by star‐branched monomer and linear crosslinker is expected to fulfill body‐temperature‐triggered softening and a lower modulus (less than 0.8 MPa) after softening.

Based on the aforementioned hypothesis, we developed a body‐temperature‐triggered in situ softening peripheral nerve electrode (BIS‐PNE). Its substrate was polymerized using the star‐branched polycaprolactone‐triol (PCL‐triol) as the monomer and the linear hexamethylene diisocyanate (HDI) as the crosslinker. The modulus of BIS‐PNE dropped from 5 MPa to only 0.64 MPa at 37 °C. The rigid BIS‐PNE provided perceptible feedback to surgeons and the softened BIS‐PNE exhibited consistent conformation to the nerve bundle. As a result, both the intraoperative injury and inflammation during long‐term implantation could be minimized. To achieve strain‐insensitivity and high‐fidelity electrical signal exchange of the BIS‐PNE, we constructed the contact electrodes by embedding gold nanotubes with a high‐aspect ratio. The contact electrodes were further modified by poly(3,4‐ethylenedioxythiophene) (PEDOT), resulting in lower interfacial impedance (325.1 ± 46.9 Ω at 1 kHz) and phase delay (0°), higher charge storage capacity (111.21 ± 5.8 mC cm^−2^) and injection capacity (3.29 ± 0.23 mC cm^−2^), compared to clinically used metallic electrodes. Benefiting from these mechanical and electrical compatibilities, the BIS‐PNE showed a favorable effect of rodentine seizure inhibition therapy with a simulation current down to 1 mA. Furthermore, an effective reduction of blood pressure and heart rate was achieved after vagus nerve stimulation (VNS) using BIS‐PNE in a hypertensive beagle model. More importantly, the alleviation of myocardial remodeling in a myocardial infarction (MI) dog was primarily realized after long‐term intermittent vagus nerve electrical stimulation with negligible inflammation. These animal experiments proved that BIS‐PNE has broad clinical application value.

## 2 Results and Discussion

### 2.1 Design and Fabrication of BIS‐PNE

**Figure 2:**
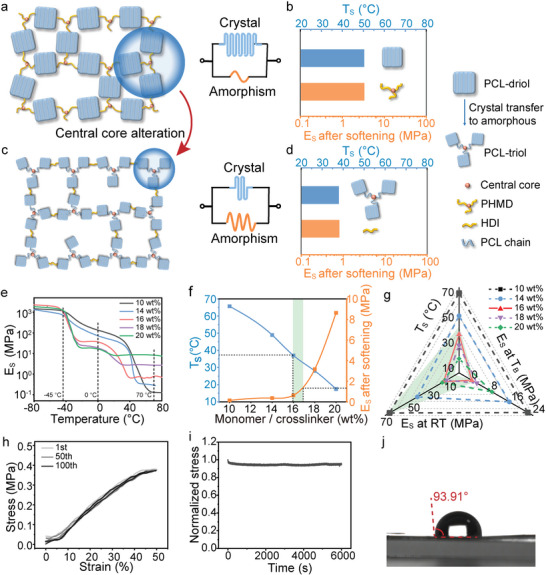
Design and fabrication of the BIS‐PNE. a) The schematic diagram of the polymer network polymerized by PCL‐diol and PHMD. b) The schematic diagram of softening temperature and storage modulus after softening of polymer polymerized by PCL‐diol and PHMD. c) The schematic diagram of the polymer network polymerized by PCL‐triol and HDI. d) The schematic diagram of softening temperature and storage modulus after softening of polymer polymerized by PCL‐triol and HDI. e) Dynamic mechanical analysis curves of the different crosslinker (HDI) ratios. f) The change curves of softening temperature and storage modulus after softening with increasing the ratio of monomer and crosslinker. g) Radar chart of the three key parameters of the electrode substrate (softening temperature, storage modulus at room temperature, storage modulus at body temperature). h) Stress–strain cycle curves of the polymer substrate. i) Stress relaxation curve of the electrode substrate at 37 °C. j) The contact angle of body‐temperature softening electrode substrate.

Peripheral nerve electrodes that possess sufficient rigidity at room temperature can provide proprioceptive feedback to surgeons, thus improving surgical success and decreasing surgical injury. More importantly, after implantation, the electrodes with a low modulus (≈0.6 MPa) have been verified to reduce the risk of inflammation.^[^ ^27^ ^]^ Thus, we designed a body‐temperature‐triggered in situ softening peripheral nerve electrode (BIS‐PNE) (Figure 1d), which consisted of a modulus transformable substrate, the strain‐insensitive Au nanotube conductive network, and the PEDOT contact electrode. The mechanical properties of our BIS‐PNE were primarily dominated by the polycaprolactone (PCL)‐based substrate, which is a widely used SMP in implantable electronics because of its intrinsic convertible modulus and biocompatibility. To our knowledge, the commonly used substrate polymerized by linear monomer (polycaprolactone‐diol, PCL‐diol) and tri‐arm crosslinker (poly(hexamethylene diisocyanate), PHMD) does not simultaneously meet the requirements of body temperature softening and nerve‐like modulus after implantation (**Figure** **2**a,b). Because there is a trade‐off between the crosslinker‐induced reduction of the crystallinity and enhancement of the crosslink density, leading to the unsolvable optimal ratio of crosslinker to monomer. As shown in Figure S1a,b (Supporting Information), the dynamic mechanical analysis curves of the films synthesized by linear monomer (PCL‐diol) and multi‐arm crosslinker (PHMD) showed that the softening temperature could be adjusted to below 37 °C by using a PHMD/PCL‐triol ratio of over 22 wt%. However, this adjustment also led to a storage modulus greater than 14.5 MPa. To address this dilemma, we replaced the PCL‐diol/PHMD with PCL‐triol/HDI (PCL‐triol with the same molecular weight as PCL‐diol), altering the central core from tri‐arm crosslinker to PCL‐triol (Figure 2c,d), which resulted in shorter arm lengths and increased binding of the central core to the arms. As a result, the competition among the crystallization of each arm was enhanced, inhibiting the regular folding and packing of the PCL chain, leading to a reduction in the crystallinity of the polymer. Furthermore, the linear crosslinker HDI effectively hindered the abrupt increase in crosslink density due to its fewer cross‐linkable terminal groups compared to the multi‐arm crosslinker PHMD. The changes in the storage modulus (E_s_) of the films with various ratios of PCL‐triol and HDI as temperature increases are shown in Figure 2e. At approximately −45 °C, a sharp decrease in the storage modulus of all samples was observed, attributed to the glass transition of the polymer films.^[^ ^28^ ^]^ At 0 °C, the storage modulus increased as the crosslinker content decreased because the crystallinity of the polymer films increased^[^ ^29^ ^]^ (Figure S2, Supporting Information). As the temperature rose, the crystalline PCL chains progressively melted, causing a further decrease in the storage modulus of the films. Once the crystals completely melt, a positive correlation between the crosslinker content and the storage modulus of the polymer films was observed, demonstrating that the crosslink density dominates the post‐softening modulus. The changes in softening temperature and storage modulus with varying crosslinker ratios are illustrated in Figure 2f. These results indicated that the substrate, fabricated by PCL‐triol and HDI, can simultaneously fulfill the criteria of body temperature softening and low modulus (0.64 MPa) after softening.

To achieve proprioceptive feedback during implantation, a substrate is required that can soften at body temperature while maintaining sufficient stiffness at room temperature (≈25 °C). To this end, we systematically screened a series of substrates with varying crosslinker contents, assessing their softening temperature, storage modulus at room temperature, and storage modulus at body temperature (Figure 2g). The goal was to identify an optimal crosslinker ratio that yields a storage modulus exceeding 5 MPa at room temperature, ensuring adequate rigidity for handling, while also exhibiting a tissue‐like modulus at body temperature to facilitate seamless integration with surrounding tissues. Our analysis revealed that a 16 wt% ratio of HDI to PCL‐triol best satisfies these criteria. This formulation provided a palpable force of ≈0.02 N to the surgeon during implantation (Figure S4, Supporting Information), enabling effective proprioceptive feedback. This optimized ratio was therefore employed in all subsequent studies unless otherwise indicated.

The electrode sites were composed of Au nanotube grafted PEDOT. Given the high aspect ratio (50–100) of Au nanotube and the robust interfacial interlock between Au nanotubes and substrate, our BIS‐PNE exhibited strain‐insensibility. As shown in Figure S5 (Supporting Information), BIS‐PNE possessed both high conductivity (1.47 × 10^4^ S cm^−1^) and only 1.9‐fold in relative resistance under 20% strain (nerve tissue strain ≈20%). PEDOT on the Au nanotubes increased the electrochemically active area, thus contributing to the high‐fidelity recording of nerve signals and low current modulation of peripheral nerves. The fabrication of the electrode was a simple in situ integration process as shown in Figure S6 (Supporting Information). To endow the BIS‐PNE with a self‐supporting helix structure, a stress‐induced thermoforming was developed (Figure S7a, Supporting Information). This geometrically customizable electrode ensured long‐term conformal contact with the nerve bundle in the absence of additional device fixation or adhesive molecule modification (Figure S7b, Supporting Information). All the merits enabled our BIS‐PNE to reduce postsurgical damage and be capable of providing a convenient surgical implantation procedure with minimal surgical damage and efficient electrical signal exchanges even under consistent motion interference.

We next characterized the resilience of the substrate, which is essential for preventing the device collapse caused by body movement and tissue locomotion during long‐term implantation. The stress–strain curve showed that the elongation at break reaches 410% (Figure S8, Supporting Information), which sufficed the maximum strain of the peripheral nerve at 20% in vivo. The modulus of the substrate at the first 100% strain was found to be 0.8 MPa, which was consistent with DMA results. Then, the cyclic tensile experiment showed the identical stress–strain curves of the 50th and 100th cycles to the first cycle. The stress relaxation experiment showed that the normalized stress remains above 95% after a sustained 100% strain for 6000 s (Figure 2h,i). These results indicated the satisfactory resilience and elasticity of the prepared polymer substrate.^[^ ^30^, ^31^ ^]^ Finally, the substrate showed hydrophobicity (Figure 2j) and a minimal swelling rate (1.73%), which were expected to maintain structural consistency during long‐term implantation in vivo.

### 2.2 Large Volumetric Capacitance

**Figure 3:**
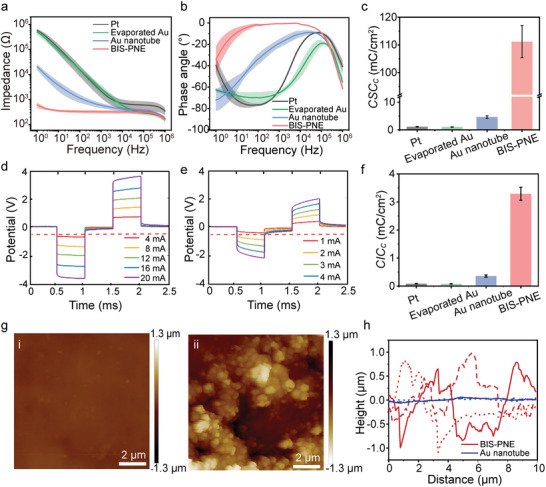
Electrochemical properties and surface roughness of our BIS‐PNE. a,b) The impedance (a) and phase angle (b) of BIS‐PNE, Pt, evaporated Au, and bare Au nanotube electrodes in the range of 1–10^6^ Hz. c) The calculated *CSC_C_* of the different electrodes according to CV curves. d,e) Voltage transients of BIS‐PNE (d) and Au nanotube electrode (e) in response to biphasic current pulses. f) Calculated *CIC_C_* of BIS‐PNE, Pt, evaporated Au, and Au nanotube electrodes according to voltage transients. g) AFM height images of Au nanotube electrode (i) and our BIS‐PNE (ii), scale bar 2 µm. h) The height curves of the Au nanotube electrode and our BIS‐PNE. Data in a, b, c, and f are presented as mean ± s.d. from 7 samples.

To evaluate the impedance and charge transfer properties of BIS‐PNE, we performed electrochemical impedance spectroscopy (EIS), cyclic voltammetry (CV), and voltage‐transient experiments in the groups of Pt, evaporated Au, and bare Au nanotube electrodes and BIS‐PNE (all with a contact electrode area of 0.06 mm^2^). The detailed testing methods are described in the Supporting Information. The EIS results showed that the impedance of our BIS‐PNE is significantly lower than that of other electrodes at frequencies below 1 kHz (**Figure** **3**a), where most physiologic signals of interest were present.^[^ ^32^ ^]^ We attribute the lower impedance of the BIS‐PNE to its highly rough morphology, which endows the interface with a highly effective surface area to enhance the volumetric capacitance.^[^ ^33^ ^]^ Meanwhile, in contrast to other electrodes, our BIS‐PNE exhibited a phase angle close to 0° in the frequency range of 1–10^5^ Hz, which signified the low phase delay during electrical signal exchanges (Figure 3b).^[^ ^34^ ^]^ The BIS‐PNE's cathodic charge‐storage capacity (*CSC*_C_), which stands for its electrochemical stability, was measured by the CV curve in the voltage window of −0.6–0.6 V^[^ ^35^ ^]^ at a scan rate of 20 mV/s (Figure S9a,b, Supporting Information). The *CSC*_C_ was calculated by the following equation:

(1) $$CSC_{C}=\int_{V_{min}}^{V_{max}}I\left(V\right)dV/2GSAs$$

where *I* is the current, *V* is the voltage window, *GSA* is the electrode geometric surface area, and *s* is the scan rate. Our BIS‐PNE exhibited more than 100‐times increase in *CSC*_C_ (111.2 ± 5.8 mC cm^−2^) compared to Pt (1.2 ± 0.1 mC cm^−2^) and evaporated Au electrodes (1.1 ± 0.1 mC cm^−2^), and 30‐times increase compared to bare Au nanotube electrodes (4.7 ± 0.5 mC cm^−2^) (Figure 3c). The large *CSC*_C_ of our BIS‐PNE may be attributed to its large electrochemically active area.

The voltage transients were measured to evaluate the cathodic charge injection capacity (*CIC*_C_), which represents the maximum amount of charge that can be injected in a current‐controlled stimulation pulse (Figure S10, Supporting Information). Biphasic current pulses were applied to the cathode with an amplitude range of 4–20 mA and pulse widths of 500 ms for both phases and pulse intervals (Figure 3d,e). The current amplitude range for the bare Au nanotube electrode was restricted to 1–5 mA. The maximum cathodic excursion potential (*E*_mc_) was measured for 10 µs following the conclusion of the cathodic current pulse. The *E*_mc_ values were then plotted as a function of the injected current amplitude, and a linear relationship was established to estimate the current limit at which the electrode would reach its cathodic voltage limit of (−0.6 V).^[^ ^32^ ^]^ The *CIC*_C_ can be calculated by the following equation:

(2) $$CIC_{C}=I_{lim}\;\times t/GSA$$

where the *I*_lim_ is the cathodic limiting current, and t is the cathodic pulse width. The results indicated that the *CIC*_C_ values (3.29 ± 0.23 mC cm^−2^) of BIS‐PNE are 40 times higher than those of Pt (0.09 ± 0.01 mC cm^−2^) and evaporated Au (0.08 ± 0.01 mC cm^−2^) electrodes and 10‐times higher than those of Au nanotube electrodes (0.36 ± 0.04 mC cm^−2^) (Figure 3f). These results suggested that our BIS‐PNE can provide a more effective charge injection than other metal electrodes at the same stimulation current.

The enhancements in electrochemical performance can be attributed to changes in surface roughness following the electrical grafting of PEDOT onto the electrode. The scanning electron microscopy (SEM) images showed that our BIS‐PNE exhibited a cauliflower morphology and porous structure (Figure S11a, Supporting Information), effectively increasing the surface roughness and electrochemically active area.^[^ ^36^ ^]^ In contrast, the bare Au nanotube electrode had a slightly rough surface (Figure S11b, Supporting Information). The height maps and 3D morphology obtained by atomic force microscopy (AFM) confirmed this observation (Figure 3g; Figure S12, Supporting Information). The height curves were further extracted from the height maps and their root mean square roughness is calculated. The results showed that the PEDOT contact electrode has a maximum height difference of 1.24 µm, which was 12.4 times higher than that of the Au nanotube electrode (0.1 µm) (Figure 3h; Figure S13, Supporting Information). Similarly, the surface roughness (353 nm) of our BIS‐PNE was 10 times higher than that of the Au nanotube electrode (35 nm) (Figure S13, Supporting Information). Due to the enhanced surface roughness and enlarged electrochemically active area, our BIS‐PNE exhibited high capacitance, resulting in lower interface impedance and phase delay, and increased charge injection capacity.

### 2.3 In Vivo Evaluation of Signal Recording and Electrical Stimulation

**Figure 4:**
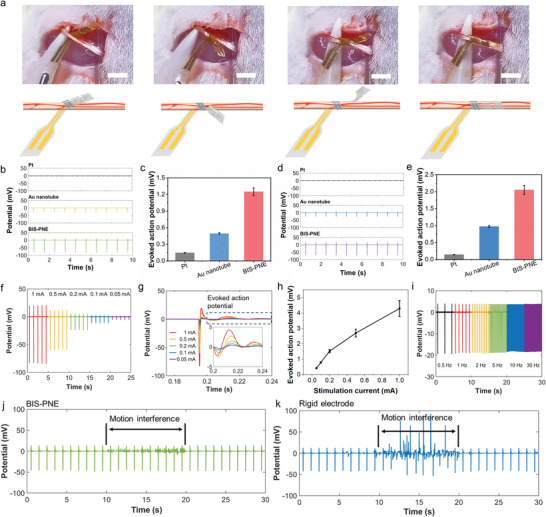
In vivo recording and stimulation performance of Pt, bare Au nanotube electrode, and BIS‐PNE. a) The optical images and diagrams of the surgical implantation procedures of our BIS‐PNE. Scale bar, 5 mm. b) Sciatic nerve action potentials recording using Pt, Au nanotube electrodes, and BIS‐PNE evoked by Pt electrode electrical stimulation. c) Evoked action potential recorded by different electrodes under 1 mA electrical stimulation. d) Sciatic nerve action potentials evoked by Pt, Au nanotube electrodes, and BIS‐PNE, respectively. e) Evoked action potentials recorded by Pt electrode under 1 mA electrically stimulated by different electrodes. f) Recorded action potentials using BIS‐PNE under varying currents stimulation evoked by BIS‐PNE. g) Enlarged view of the comparison between the five evoked action potentials. h) The amplitude of evoked action potentials under different current stimulation. i) Recorded action potentials evoked by 0.2 mA stimulation in the frequency range of 0.5–30 Hz. j,k) Sciatic nerve action potentials recording by a BIS‐PNE (j) and a rigid electrode (k) under motion interference. Data in c, e, and h are presented as mean ± s.d. from 7 samples for c and e, and 5 samples for h.

To demonstrate the ability of our BIS‐PNE to record nerve signals and deliver electrical stimulation, we wrapped the BIS‐PNE (contact electrode sized at 0.6 mm width), bare Au nanotube, and Pt electrodes around the rat sciatic nerve. The implantation process of the BIS‐PNE is presented in **Figure** **4**a. Taking advantage of the shape forming in advance, the BIS‐PNE could be easily and tightly twined around the sciatic without additional mechanical locking. To compare the recording and stimulating performance of BIS‐PNE, we used BIS‐PNE, Au nanotube electrode, and Pt electrode as stimulation or recording electrodes for testing. We delivered a monophasic square wave with a frequency of 1 Hz and 500 µs pules width to the sciatic nerve through a Pt electrode. Notably, the compound action potentials recorded by BIS‐PNE under 1 mA current stimulations displayed significantly higher amplitude (2.05 ± 0.14 mV) than those recorded by Au nanotube electrode (0.97 ± 0.02 mV) and Pt electrode (0.15 ± 0.01 mV) (Figure 4b,c). This high‐fidelity recording of the electrical signal was attributed to the lower interfacial impedance of BIS‐PNE. Thus, our BIS‐PNE can sensitively capture subtle electrical signals of nerves. Additionally, to evaluate the stimulating property of our BIS‐PNE, we used a Pt electrode to record the compound action potentials evoked by BIS‐PNE, Au nanotube, and Pt electrodes (electrical stimulation 1 mA). The results show that the evoked action potentials (1.2 ± 0.30 mV) by BIS‐PNEs were higher than those of Pt electrodes (0.15 ± 0.01 mV) and Au nanotube electrodes (0.57 ± 0.12 mV) (Figure 4d,e), which could be attributed to the lower interface impedance and higher charge injection capacity of our BIS‐PNE. It is worth noting that the action potential evoked by BIS‐PNE at 0.1 mA current stimulation is approximately equal to that of the Pt electrode at 1 mA stimulation (Figure S14, Supporting Information). This attribute reduces stimulation current and, consequently, stimulation injury.

Furthermore, the BIS‐PNE could serve as a stimulation and a recording electrode to explore its potential applications. We delivered a series of currents ranging between 0.05 and 1 mA to the sciatic nerve using the BIS‐PNE. As shown in Figure 4f, the corresponding compound action potentials recorded by BIS‐PNE are identical for each current stimulation, indicating its stable and reliable exchanges of the electrical signal. We further compared the evoked compound action potentials under different current stimulations, which revealed consistent waveforms but with increasing peak potentials with increasing current stimulation (Figure 4g,h). We also explored the frequency range of electrical stimulation and signal recording at a stimulation current of 0.2 mA. The results show that the BIS‐PNE displayed a broad frequency range (0.5–30 Hz) (Figure 4i), which is appropriate for treating various refractory diseases, such as epilepsy and heart failure therapy.^[^ ^37^, ^38^ ^]^

Involuntary physical shakes can occur in refractory diseases such as seizures and Parkinson's disease. A robust electrode‐nerve interface is necessary for delivering electrical stimulation and recording peripheral nerve signals under violent body movement. We applied a 1 mA current stimulation with a frequency of 1 Hz to the sciatic nerve and recorded the action potential using BIS‐PNEs to determine whether our BIS‐PNE can resist motion interference and subsequently compare the results with rigid electrodes (Figure 4j,k). The rigid electrodes were constructed using a non‐softening substrate (≈100 MPa) and are fabricated in the same manner as BIS‐PNE electrodes. The signals recorded by BIS‐PNE exhibited consistent waveforms with or without motion interference, despite slight noise (RMS noise 0.12 mV). In contrast, the rigid electrode recorded action potentials with such distortion that we are unable to recognize normal signal waveforms (Figure 4k, RMS noise 0.46 mV). According to the observation of the interface between the electrode and nerve, we attribute this dramatic distortion to the shaky interface that generates gaps between the rigid electrodes and the sciatic nerve during body movement (Figure S15, Supporting Information). Therefore, our BIS‐PNE can maintain a robust interfacial connection and electrical signal exchanges during body movement.

### 2.4 VNS for In Vivo Neuromodulation

**Figure 5:**
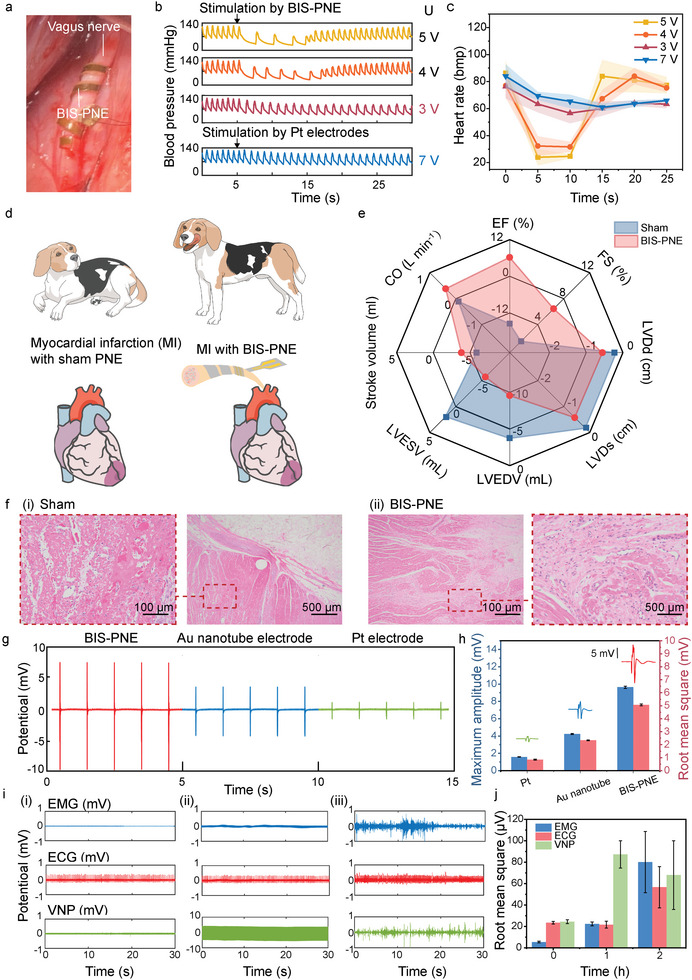
Neuromodulation reduces blood pressure, alleviates myocardial remodeling, and inhibits epileptic seizure by VNS using BIS‐PNE. a) The optic image of BIS‐PNE which is compatibly wrapped around a vagus nerve. b) The real‐time monitoring of blood pressure when stimulated with Pt electrode and BIS‐PNE in various voltages. c) The changes in heart rate when stimulated with Pt electrode and BIS‐PNE in various voltages (n=3). d) The dogs for MI modeling and treatment were divided into a sham group and a BIS‐PNE group. e) The changes in EF, FS, left ventricular end‐diastolic diameter (LVDd), left ventricular end‐systolic diameter (LVDs), LVEDV, LVESV, Strock volumes, and CO between postoperative day 3 and day 15 (n = 3). f) Results of HE staining in infarct areas of sham (i) and BIS‐PNE (ii) groups after treatment for 15 days. (a–d) Muscle probes recorded EMG signals evoked by BIS‐PNE, Pt electrode, and Au nanotube electrode. And the enlarged views were shown at the top of the respective group in (h). h) The maximum amplitude and root mean square of evoked EMG signals. i) Experiment group EMG, ECG, and VNP after injection pilocarpine 0 h (i), 1 h (applying VNS treatment) (ii), and 2 h (iii). j) The root mean square of three electrical signals (experimental group). Data in h and j are presented as mean ± s.d. from 5 samples.

To validate the therapeutic efficacy of using BIS‐PNE in VNS, we implanted it into the right vagus nerve of hypertensive beagle dog models (**Figure** **5**a). For the canine experiments, the contact electrodes of BIS‐PNEs were constructed to a precise width of 1 mm (Figure S16, Supporting Information). The softened BIS‐PNE exhibited an ideal fit with the canine vagus nerve. Therefore, only a voltage of 3 V was required to achieve progressive negative inotropic and lusitropic responses by BIS‐PNEs for VNS (Figure 5b,c). Excessive voltages (5 and 4 V) led to abrupt reductions in blood pressure and heart rate, inducing deleterious effects of VNS, such as bradycardia. To achieve similar responses with platinum (Pt) electrodes, a voltage of up to 7 V was necessary, implying greater energy consumption. At voltages of 8 and 6 V, the modulation effects demonstrated overstimulation and insufficient stimulation, respectively (Figure S17, Supporting Information). After obtaining the reasonable parameter of stimulation voltage, we applied BIS‐PNEs to the treatment of myocardial infarction (MI).

The chronic sympathetic nervous system excitation post‐MI accelerates the loss of cardiac function, ultimately leading to heart failure in patients.^[^ ^39^ ^]^ We constructed MI beagle dog models by ligating the left anterior descending coronary artery (Figure S18, Supporting Information). The noticeable elevation of the S‐T segments in real‐time electrocardiography (ECG) indicated successful modeling of MI^[^ ^40^ ^]^ (Figure S19a, Supporting Information). The dogs in the sham group underwent identical surgical procedures as those in the BIS‐PNE group but did not receive VNS treatment (Figure 5d). As shown in Figure 5e, after 15 days of VNS, the dogs in the BIS‐PNE group exhibited significant cardiac functional improvements. Figure 5e summarizes the changes in the echocardiography between postoperative day 3 and day 15. The ejection fraction (EF) in the BIS‐PNE group remained within the normal range (> 50%), while in the sham group, EF decreased from 61.4 to 45.9%. Clinically, EF below 50% is usually associated with heart failure due to decreased cardiac output.^[^ ^41^ ^]^ Additionally, in the BIS‐PNE group, left ventricular fractional shortening (FS) increased from 24.6 to 31.2%, cardiac output (CO) increased from 1.3 to 1.7 L min^−1^, left ventricular end‐diastolic volume (LVEDV) decreased from 28.1 to 18.8 mL, and left ventricular end‐systolic volume (LVESV) decreased from 12.9 to 7.46 mL. These commonly used indices for evaluating cardiac pump function indicated cardiac dysfunction in the sham group.^[^ ^42^, ^43^ ^]^ However, these parameters of the sham group exhibited a contrary tendency compared with the BIS‐PNE group, indicating the typical course of heart failure. Histological examination explains the reasons for these differences. Fibrous protein deposition, patchy myocardial fiber necrosis, dissolution, and vacuolization were extensively observed in the infarcted myocardium and border zone in the sham group (Figure 5f (i)). These histopathologic evolutions were consistent with other research.^[^ ^44^ ^]^ In contrast, the BIS‐PNE group showed only localized patchy myocardial fibrosis with sparse lymphocytic infiltration (Figure 5f (ii)). This attenuating effect of VNS on abnormal myocardial matrix remodeling was a primary reason for protecting the infarcted heart from ventricular tachycardia (VT).^[^ ^45^ ^]^ The real‐time ECG confirmed the decreased incidence of VT in the BIS‐PNE group compared to the sham group (Figure S19b,c, Supporting Information). These results demonstrated that our BIS‐PNE electrodes achieve reliable improvements in cardiac function and myocardial remodeling by VNS with reasonable voltage. This treatment effectiveness in large animal models underscores the significant potential of our BIS‐PNE as a physical therapeutic medical device for cardiovascular diseases.

VNS has been clinically approved to be used for patients with drug‐resistant epilepsy.^[^ ^46^ ^]^ We first utilized BIS‐PNE, bare Au nanotube electrode, and Pt electrode to stimulate the rat sciatic nerve with a current density of 0.5 mA. Meanwhile, the muscle probe electrodes are inserted at the corresponding locations in the hind leg to record the electromyography (EMG) (Figure 5g). For the rat experiments, the electrodes are 0.6 mm in width (Figure S7, Supporting Information). The results indicated that stimulation using BIS‐PNE generates the highest signal amplitude (9.8 mV), in contrast to the Au nanotube electrode (4.3 mV) and Pt electrode (1.5 mV). The root mean square (RMS) values of myoelectric signals follow a similar trend (Figure 5h). These results reconfirmed the superior charge injection capacity of our BIS‐PNE and suggested its potential to repair damaged nerves and alleviate muscular dystrophy with lower current intensity.^[^ ^47^, ^48^ ^]^ Then, the therapeutic effect of VNS using the BIS‐PNE in an anesthetized rat model of epilepsy is tested. ECG, EMG of the left forelimb, and vagus nerve potentials (VNP) are simultaneously detected in epileptic seizures (Figure S20, Supporting Information). Pilocarpine (10 mg kg^−1^ at 10 min intervals) was injected to induce epileptic seizures. EMG, ECG, and VNP signals of rats are recorded (Figure 5i (i); Figure S21a, Supporting Information), followed by batch injections of pilocarpine until the seizures. In the experimental group, electrical stimulation of 1 mA at 25 Hz was applied to the left vagus nerve following pilocarpine injection, while no stimulation was administered in the control group. The seizures occurred ≈1 h after the initial pilocarpine injection, accompanied by generalized spasticity and shivering in the control group. As a result, the seizure‐induced abnormal EMG, ECG, and VNP are observed (Figure S21b, Supporting Information). The body spasticity and shivering caused by abnormal neuronal discharge induced the enhancement of RMS values. These abnormal signals persisted during the 2 h after injection of pilocarpine in the control group (Figure S21c, Supporting Information), which indicated continual seizures. The control group's RMS of these electrical signals is shown in Figure S18d (Supporting Information). Contrary to the control group, the abnormal EMG caused by body spasticity and shivering disappeared in the experiment group and the ECG remains consistent with the signals obtained before seizures (Figure 5i (ii)). The VNP during this period was the current stimulus‐induced compound action potentials instead of abnormal signals (Figure S22, Supporting Information). Interestingly, when 2 h of stimulation of the left vagus nerve stopped, the EMG, ECG, and VNP signals returned to the abnormal state which were like those of the control group after seizures (Figure 5i (iii)). The experiment group's RMS of these electrical signals is shown in Figure 5j. The large error bars in Figure 5j at the 2 h mark is due to significant signal variations, such as transient spikes, which naturally contribute to increased variance. To capture overall trends and minimize the impact of noise, the RMS values were calculated from the signal envelope. Despite the variability, the data offered valuable insights into the physiological dynamics under investigation. Additionally, it is important to note that the control group also exhibited similarly large error bars at the 2 h mark, further emphasizing the inherent variability at this time point. These results indicated that our BIS‐PNE successfully suppresses the development of epileptic seizures by delivering low current stimulation (1 mA) to the vagus nerve.

### 2.5 Biocompatibility after Chronic Implantation

**Figure 6:**
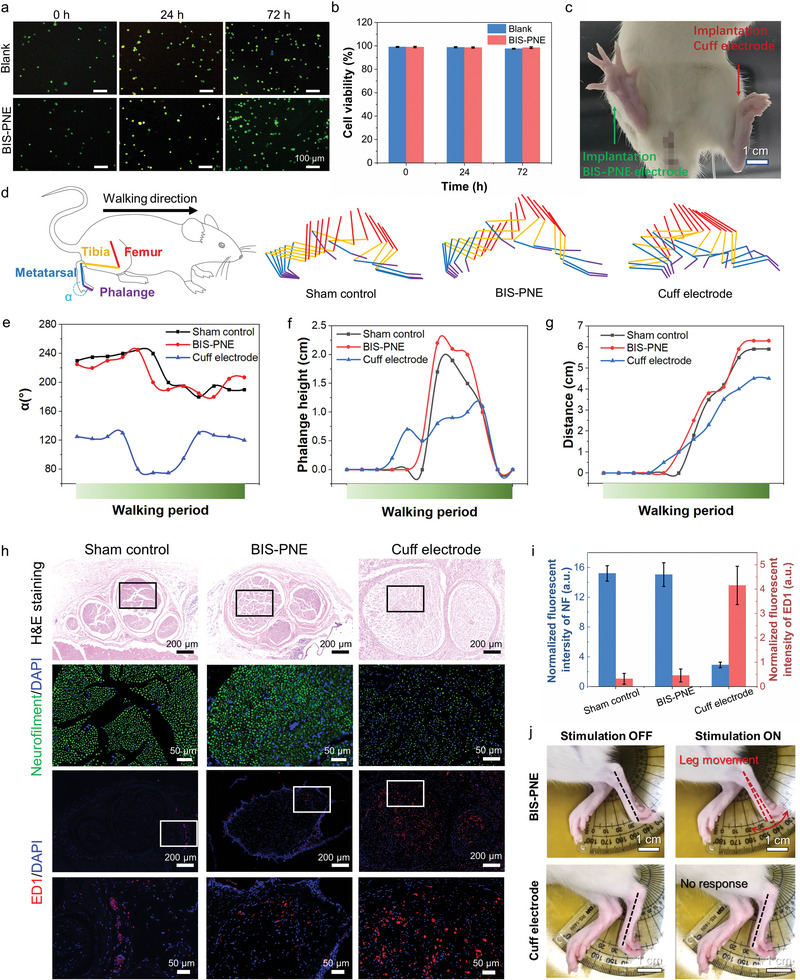
The biocompatibility and behavior study of BIS‐PNE on sciatic nerves. a) Microscopic images of 3T3 cells at 0, 24, and 72 h culture with blank (the first row) and BIS‐PNE (the second row). b) Cell viability comparison of blank and BIS‐PNE groups at different culture times. c) The photograph of hindleg implantation with BIS‐PNE and cuff electrode. d) Hindleg stick diagram and comparison of kinematic features during walking after implantation 4 weeks with the sham control, BIS‐PNE, and cuff electrodes. e–g) The behavior parameters during walking, including angle (α) between metatarsal and phalange (e), phalange height (f), and walking distance (g). h) Cross‐sectional slices of the sciatic nerve were implanted with the sham control, the BIS‐PNE, and the cuff electrode. H&E staining (the first row), fluorescent images immunochemically labeled by the biomarker neurofilament (NF) and 4′,6‐diamidino‐2‐phenylindole (DAPI) (the second row), fluorescent images immunochemically labeled by the biomarker ED1 and DAPI (the third row), and magnified images (the fourth row). i) The normalized fluorescence intensity of NF and ED1 for sham control, BIS‐PNE, and cuff electrode groups. j) Optical images of leg movement under sciatic stimulation after implantation of BIS‐PNE and cuff electrode for 4 weeks at 0.5 mA, 1 Hz. Data in b and i are presented as mean ± s.d. from 7 samples.

The live/dead assay and cell counting kit‐8 (CCK‐8) assay were performed to evaluate the cellular compatibility of our BIS‐PNE. Given that the peripheral nerve sheath is composed of fibroblasts, and in light of previous studies utilizing 3T3 cells for peripheral nerve electrode evaluation,^[^ ^49^, ^50^ ^]^ we selected the fibroblastic 3T3 cells to validate the in vitro biocompatibility of our BIS‐PNE. After culturing 3T3 cells with BIS‐PNE for 0, 24, and 72 h, the density of live cells (green fluorescence) was extremely close to the blank group (**Figure** **6**a), and the statistical result is shown in Figure 6b. Furthermore, the results of the CCK‐8 assay were consistent with the live/dead assay experiment (cell viability over 98% after 72 h) (Figure S23, Supporting Information). These results demonstrated that our BIS‐PNE possesses cell safety.

The mechanical mismatch between implantable devices and tissues can potentially generate inflammatory responses. To assess the chronic implantation biosafety of BIS‐PNE, it was twined around the sciatic nerve in the left hindleg of the rats for 4 weeks while allowing the rats to move freely (Figure 6c). Meanwhile, flexible cuff electrodes (modulus ≈2.4 GPa) made of perylene and a thin film of platinum were also implanted on the right hindleg of the rats.^[^ ^51^ ^]^ The flexible cuff electrodes used for comparison are commercial Pt electrodes, thus ensuring a fair evaluation. Compared to the sham control, our BIS‐PNE had no significant impact on the movement function of the rats. Specifically, their left hindlegs showed normal toe spread and similar angles (α) between the metatarsal and phalange, walking displacement, and limb height during one walking period (Figure 6d–g). In contrast, the implantation of cuff electrodes resulted in serious limb dysfunction, including whole‐foot placement on the ground and foot‐dragging during ambulation.

To better understand the cause of sciatic nerve dysfunction, we examined the histological changes in the sham control, BIS‐PNE, and cuff electrode groups at 4 weeks after implantation. Based on H&E staining, no histopathological changes were observed in the BIS‐PNE group when compared with the sham control. However, the cuff electrode group showed several areas of edema (appearing as lighter areas compared to the sham control) (Figure 6h, the first row).^[^ ^52^, ^53^ ^]^ Similarly, the levels of neurofilament staining (Figure 6h, the second row) and the expression levels of ED1 (a pan‐macrophage marker in rats) (Figure 6h, the third and the fourth rows) were not significantly different between the sciatic nerve tissues of the BIS‐PNE implanted group and the sham control. In contrast, the expression levels of neurofilaments in the cuff electrode group were exceedingly lower compared to the sham control, while ED1 exhibited a higher expression (Figure 6i).^[^ ^54^ ^]^ The experimental group implanted with rigid electrodes also showed tissue edema and higher levels of inflammatory response than the BIS‐PNE group (Figure S24, Supporting Information). Furthermore, periodic hind leg movements (≈5°) were observed when using BIS‐PNE with electrical stimulation at 200 µA,1 Hz after the 4 weeks of implantation. However, there was no response observed for the cuff electrode under the same stimulation conditions (Figure 6j). The results of the combined gait and histopathology analysis suggested that our BIS‐PNE can be chronically implanted without causing negative impacts on peripheral nerves and surrounding tissues.

## 3 Conclusion

We demonstrate the feasibility of a modulus‐convertible peripheral nerve electrode (BIS‐PNE) fabricated via a simple planar processing technology using star‐branched PCL‐triol and a linear crosslinker. This unique design allows the BIS‐PNE to conform to the nerve interface and soften in situ at body temperature, ensuring a robust and stable bio‐interface for long‐term implantation. Importantly, the BIS‐PNE provides valuable proprioceptive feedback to surgeons during implantation, minimizing mechanical constraints. The helical BIS‐PNE exhibits high conductivity and strain insensitivity due to its Au nanotube conductive network, enabling high‐fidelity nerve signal recording and low‐current nerve modulation. Furthermore, the increased electrochemical active area enhances volumetric capacitance, further improving the electrodes’ performance.

Adequate long‐term in vivo neuromodulation experiments demonstrate the significant therapeutic potential of our BIS‐PNE. Programmed electrical stimulation via the BIS‐PNE successfully preserves cardiac ejection function in MI dogs and mitigates undesirable cardiac remodeling. Additionally, BIS‐PNE successfully suppresses seizures in rats. There are no adverse effects on the neural tissue and neural function of the rats caused by our BIS‐PNE after 4 weeks of implantation. This combination of features highlights the potential of the BIS‐PNE to advance the field of neural interfacing and improve clinical outcomes for a wide range of neurological conditions.

## 4 Experimental Section

### Materials

Silver nitrate (AgNO_3_) and ethylenediamine (en) were purchased from Sinopharm chemical reagent Co., Ltd. Polyvinylpyrrolidone (PVP, Mn 360 k), copper chloride (CuCl_2_∙2H_2_O), ethylene glycol, Sodium tetrachloroaurate dihydrate (NaAuCl_4_∙2H_2_O), caprolactone, trimethylolpropane, stannous isocaprylate (SnOct_2_), butyl acetate, hexamethylene diisocyanate (HDI) were obtained from Aladdin biochemical technology Co., Ltd.

### Synthesis of the Polycaprolactone‐Triol

The PCL‐triol was synthesized by ring‐opening polymerization of caprolactone. Briefly, 20 g of caprolactone and 1.208 g of trimethylolpropane were added into a double neck flask and heated to 120 °C under continuous N_2_ purge and magnetic stirring. After trimethylolpropane was dissolved completely, 70 µL stannous isocaprylate (SnOct_2_) was dropped, and the reaction was maintained for 24 h. as‐synthetic polycaprolactone triol (PCL‐triol) was purified by precipitation in methanol and drying under vacuum for 12 h at 60 °C. The molecular weight was determined by Gel Permeation Chromatography (GPC, Agilent waters 1515) to be 3732 g mol^−1^, and the molar mass dispersity was 1.27. H^1^ nuclear magnetic resonance (NMR) spectra of synthetic PCL‐triol show that the resonance peak appearance at δ = 3.7 ppm represents the hydroxyl end‐groups of PCL‐triol (Figure S25, Supporting Information), indicating that PCL‐triol is successfully synthesized.

### Preparation of the Silver Nanowires (AgNWs)

The AgNWs were prepared by polyol process according to the previous report. 380 mg PVP was added into 130 mL of ethylene glycol and pre‐heated at 175 °C under magnetic stirring (500 rpm). After the PVP was dissolved, 800 µL 4 mM CuCl_2_∙2H_2_O was added. After stirring 10 min continuously, the 30 mL of 0.095 M AgNO_3_ was dropped in 10 min. After finishing the drop, the stirring was stopped, and the preparation reaction was maintained for 30 min. The prepared AgNWs were diluted with 640 mL of water and were washed multiple times using water and ethanol. Last, the prepared AgNWs were redispersed in water at ≈5 mg mL^−1^.

### Synthesis of the [Au(en)_2_]Cl_3_

The [Au(en)_2_]Cl_3_ was synthesized according to the reported literature. Briefly, 1.6 mL of 1.0 m an aqueous solution was dropped into 8 mL of 0.1 m NaAuCl_4_·2H_2_O under magnetic stirring at room temperature. After stirring for 30 min continuously, the undissolved precipitate was removed by centrifuge, and the yellow liquid supernatant was added to the 200 mL of ethanol. The pale‐yellow precipitate was separated, collected by centrifuge, and dried under vacuum at room temperature.

### Preparation of the Gold Nanotubes (AuNTs) Conductive Layer

0.8 mL AgNWs dispersion with a concentration of 5 mg mL^−1^ was dropped to the glass plate mold and dried at room temperature as AgNWs film. The synthesized [Au(en)_2_]Cl_3_ was dissolved in deionized water with a concentration of 1 mM. To synthesize the AuNTs conductive layer, the AgNWs film was soaked into 25 mL of prepared [Au(en)_2_]Cl_3_ solution and reacted for 12 h at 90 °C. After the reaction, the AuNTs conductive layer was transferred into 20 wt% nitric acid solution and 10 wt% ammonium hydroxide successively at 90 °C to remove unreacted silver and precipitated AgCl.

### Preparation of the Peripheral Nerve Electrode

A 5 wt% polyvinyl alcohol (PVA) solution was dropped into the AuNTs conductive layer. Amounts of PVA were distributed in the interface of the glass mold and conductive layer when the water evaporated. 0.4 g of synthesized PCL‐triol and 0.1 g of PCL‐diol were added into a glass bottle and melted at 100 °C. After melting, 700 µL butyl acetate and 70 µL hexamethylene diisocyanate (HDI) were added under tempestuous stirring. After stirring for 5 mins, 3.7 µL of SnOct_2_ was added and persistently stirred for 3 min as a precursor. Then, the 200 µL precursor was dropped to AuNTs conductive layer, cured at 60 °C for 2 h, and dried under vacuum at 80 °C for 2 h as a body temperature soft substrate. When the substrate was cured, the AuNTs conductive layer was embedded in the surface of the substrate. The patterning of the conductive layer was produced using a laser process and encapsulated using parylene‐C. A laser marking machine (Model: SX‐OEM1, Shenzhen Yingnuo) was usedfor the laser machining process. The parameters for the laser machining include a laser power of 35%, a speed of 500 mm^−1^s^−1^, and a frequency of 50 kHz. The contact electrode of the peripheral nerve electrode was modified using PEDOT by in‐site electrical grafting. Finally, the peripheral nerve electrode was endowed spiral by fixed on a capillary and heated to 180 °C for 10 mins.

### Chemical and Morphological Characterization

Fourier‐transform infrared spectroscopy (FT–IR) was recorded on Horiba HR Evolution. The X‐ray diffractograms (XRD) were performed on Bruker D8 Advance with a wide angle (5–80°). SEM images were captured using a HITACHI SU8010 scanning electron microscope. AFM images were collected by Bruker Dimension ICON with tapping mode. The contact angle of the softenable polymer substrate was measured using a Dataphysics OCA20 Contact Angle/surface tension tester.

### Measurements of Electrical and Mechanical Properties

The conductivity of the Au nanotube conductive layer was measured through the four‐point probe method with a Keithley 2450 source meter. The width and length of the conductive layer were obtained using a vernier caliper. The relative resistance changes of the Au nanotube and evaporated Au nanofilm were measured using a Keithley 2450 source meter and Criterion Electromechanical Test Systems (C42.503, MTS) at a tensile speed of 10 mm min^−1^. The stress–strain, stress relaxation, and tensile cycle curves at 100% strain of softenable polymer substrate were measured by Criterion Electromechanical Test Systems at a tensile speed of 10 mm min^−1^. The changes of storage modulus with temperature (−100–100 °C) at different ratios of crosslinker were measured using dynamic mechanical analyses (DMAs; Q800, TA Instruments) under “multifrequency, strain” mode at 1 Hz, 0.1% strain, and a heating rate of 3 °C min^−1^.

### Electrochemical Characterization

Electrochemical measurements, including EIS, CV, and current pulsing, were performed for planar BIS‐PNE, Pt, evaporated Au, and Au nanotube electrodes with an area of 0.6 mm^2^ in 10 mm phosphate buffered saline (Quality Biological, pH 7.4) using an AMS Model 4100 Isolated High Power Stimulator. Pt electrodes were fabricated by embedding 0.1 mm Pt wires on parylene films and subsequently encapsulating these wires in parylene with a 2 mm opening pre‐cut to form the electrode. Evaporated Au electrodes were fabricated by depositing 10 nm Ti followed by 400 nm Au on the polymer substrate and subsequently encapsulating these films using parylene by chemical vapor deposition (CVD) process. Au nanotube electrodes were fabricated by a similar method as BIS‐PNE except by electrical grafting conductive polymer. All electrochemical measurements were performed in a three‐electrode configuration with a Pt wire counter electrode and an Ag/AgCl reference electrode. EIS was measured from 1 to 10^6^ Hz with a 10‐mV driving voltage. Cyclic voltammetry was performed at a sweep rate of 20 mV s^−1^. Voltage‐transient experiments were measured by using the constant current stimulator and oscilloscope.

### In Vivo Animal Experiment

The animal experiments were performed in the Animal Core Facility of Nanjing Medical University and approved by the Institutional Animal Care and Use Committee of Nanjing Medical University (IACUC‐2205049).

### Experiments in the Hypertensive Dog Model

The animals were induced with Zoletil (dose 0.1–0.5 mL kg^−1^) for anesthesia, followed by mask inhalation of isoflurane and intubation with continued isoflurane administration through the endotracheal tube. Then, the femoral artery of the hind limbs was cannulated for invasive blood pressure monitoring. The ECG was recorded as well. A non‐invasive blood pressure monitoring device was used on the opposite femoral artery to collect radial artery pulse signals and ECG. Additionally, VNE signals and sympathetic nerve signals were recorded using BIS‐PNE.

Intravenous injection of norepinephrine‐induced a significant and stable increase in blood pressure. After implanting BIS‐PNE, electrical stimulation was administered using parameters of either a single‐phase wave for 0.1 ms or a biphasic wave for 0.2 ms, at frequencies ranging from 2 to 15 Hz, and amplitudes varying from 1 to 3 mA. Stimulation was applied continuously or intermittently.

### Experiments in the MI Dog Model

After standard gas anesthesia, assisted ventilation was established. A 3.0 cm incision was made at the third intercostal space along the left sternal border. Titanium clips were used to secure the left anterior descending coronary artery of the dog. S–T segment elevation was observed on the electrocardiogram, confirming successful modeling. Finally, the left anterior descending coronary artery was ligated using suture threads.

The VNS was administered using parameters of a frequency range from 20 to 30 Hz, pulse width of 250 ms, and current intensity ranging from 0.5 to 2 mA. Each stimulation session lasted 30 min, administered twice daily with an 8 h interval, continuously for 15 days. The sham group did not receive VNS treatment.

### Sciatic Nerve Electrical Stimulation and Signal Recording

Sprague Dawley (SD) rat was anesthetized by inhalation of isoflurane (5%)/air, and anesthesia was maintained with isoflurane (2%)/air. A 2‐cm incision of the skin was made, and the sciatic nerve was exposed by separating muscles close to the femur. BIS‐PNE, Pt, evaporated Au, or Au nanotube electrodes were placed on the exposed sciatic nerve for electrical stimulation or signal recording. For comparison of the performance of signal recording, the Pt electrode was the stimulation electrode, and another Pt electrode, Au nanotube electrodes, and BIS‐PNE serve as the recording electrode. For comparison of the performance of electrical stimulation, the Pt electrode was the recording electrode, and another Pt, Au nanotube electrodes, and BIS‐PNE serve as the stimulation electrode. Various current amplitudes (from 50 µA to 1 mA, 1 Hz) were applied by a constant current stimulator. The evoked compound action potentials were recorded using a Differential AC Amplifier (1700, A‐M System) and PowerLab (PL3516, AD Instruments).

### Vagus Nerve Electrical Stimulation Inhibition Epileptic Seizure

SD rat was anesthetized by inhalation of isoflurane (5%)/air, and anesthesia was maintained with isoflurane (2%)/air. A 2 cm incision of the skin was made on the left neck, and the vagus nerve was exposed by separating muscles. Two BIS‐PNEs were twined on vagus nerves as stimulation electrodes and recording electrodes, respectively. Meanwhile, ECG and EMG of the left forelimb were recorded. Next, pilocarpine was injected in batches until seizures, 10 min apart, and 10 mg kg^−1^. For the control group, no VNS and the various signals were recorded for 2 h. For the experimental group, VNS started with the first injection of pilocarpine, 1 mA 25 Hz. The ECGs of rats were recorded by attaching electrodes to the right and left front paws as cathode and anode and an electrode to the right hind foot as reference. EMG signals were recorded by inserting three needle electrodes into the muscles of the left forelimb as the anode, cathode, and reference, respectively. VNP was monitored by twisting the BIS‐PNE on the left vagus nerve near the stimulation electrode. The BIS‐PNEs were prepared with an inner diameter of 0.5 mm, corresponding to the diameter of the vagus nerve of the rat. BIS‐PNEs can form a conformal interface with the vagus nerve of rats, which facilitates charge transfer between electrodes and nerves. An animal model of rat epilepsy was prepared by injection of pilocarpine in batches. For the control group, the EMG, ECG, and VNP signals of a rat were recorded.

### In Vitro Biocompatibility

The in vitro biocompatibility of BIS‐PNE was verified by live/dead cell staining with 3T3 mouse embryo fibroblast cells. The BIS‐PNEs were soaked in 75% alcohol for 1 h and following washed with PBS several times to remove residual alcohol. The BIS‐PNE and cells (300000 mL^−1^) were co‐cultured in a sex‐well culture plate. The cells were stained with calcein‐AM and propidium iodide (PI) after 0, 24, and 72 h culturing, and their morphology and proliferation were examined by fluorescence microscopy. The cell counting kit‐8 (CCK‐8) assay was also performed to measure cell viability.

### Histological Analysis, Immunostaining, and Behavioral Analysis

All four rats were used to analyze the effect of chronic implantation (4 weeks) with sham, BIS‐PNE, cuff electrode (KD‐cuff‐2, Kedou BC), and without softening electrode. A video camera to record the trajectory of the legs of the rats during walking to analyze the effect of electrode implantation on leg function. Subsequently, the rats were euthanized with isoflurane (5%)/air gas mixture. The sciatic nerve and surrounding muscle tissues were sampled and fixed with 4% paraformaldehyde for 24 h. Next, the samples were embedded in paraffin and cut at 5 mm, and deparaffinized by immersing xylene. Last, the samples were stained (H&E and immunofluorescence) using standardized histological procedures. The quantitative evaluation of fluorescence intensity was using image J software.

## Conflict of Interest

The authors declare no conflict of interest.

## Author Contributions

X.R. and W.T. equally contributed equally to the work. X.R., W.T., and B.H. designed the project. W.T. fabricated the BIS‐PNEs used in this work. X.R., W.T., M. C. designed the animal experiments, and W.T., S.C., J. M., Y.Y., J. F., and X. F. conducted the relative experiments. X.R. and W.T. wrote this part of the paper. W.T. and F.L. conducted the in vitro cytotoxicity test and wrote this part of the paper. X.R., W.T., and B.H. wrote the paper. All authors read and revised the paper.

## Supporting information



Supporting Information

Supplemental Movie 1

## Data Availability

The data that support the findings of this study are available from the corresponding author upon reasonable request.
