# Supplementary material for: A Body‐Temperature‐Triggered In Situ Softening Peripheral Nerve Electrode for Chronic Robust Neuromodulation
Source: Adv Sci (Weinh). 2024 Dec 6;12(5):2412361. doi: 10.1002/advs.202412361 (PMC11791928; doi:10.1002/advs.202412361)
Supplement: Supplementary file 1 — Supporting Information [file ADVS-12-2412361-s002.docx]

Supporting Information

A Body-temperature-triggered in-situ Softening Peripheral Nerve Electrode for Chronic Robust Neuromodulation

Xueyang Ren, Wenjie Tang, Yuehui Yuan, Shisheng Chen, Fangzhou Lu, Jinyang Mao, Jidan Fan, Xufeng Wei, Ming Chu*, Benhui Hu*


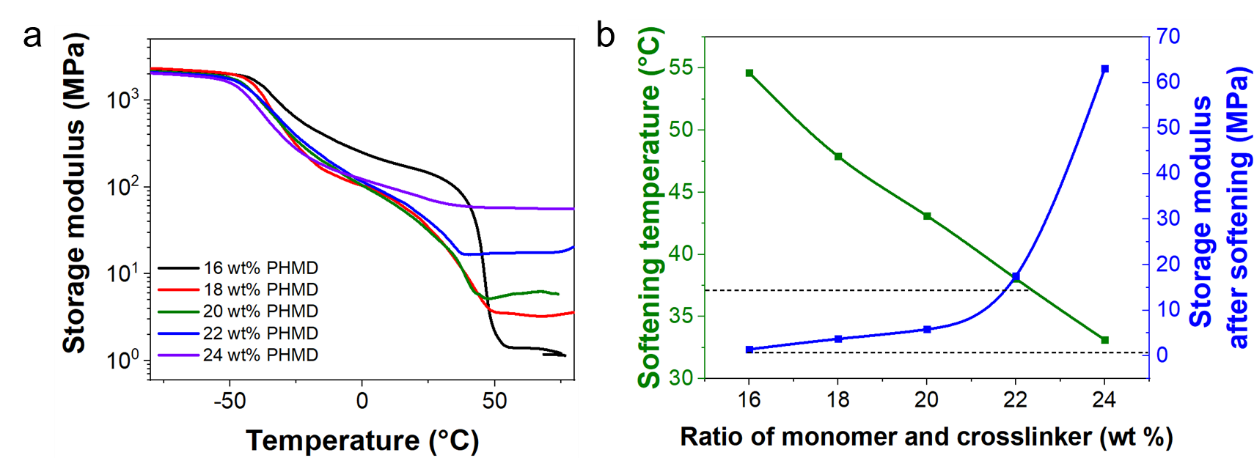


Figure S1. Dynamic mechanical analysis curves of the different ratios of PCL-diol/PHMD.


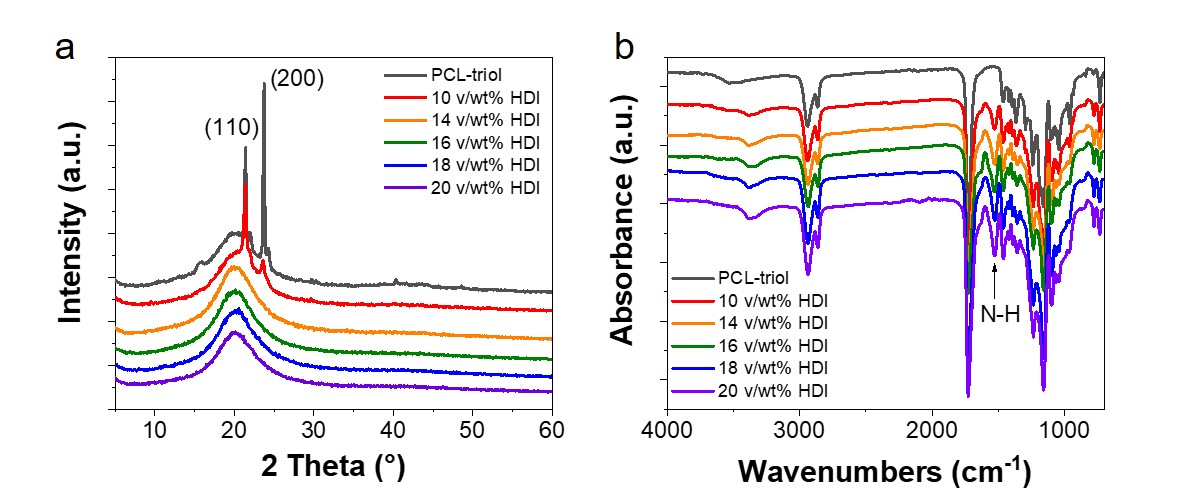


Figure S2. XRD (a) and FT-IR Spectroscopy (b) of pure PCL-triol and polymer films prepared by different ratios of crosslinker.


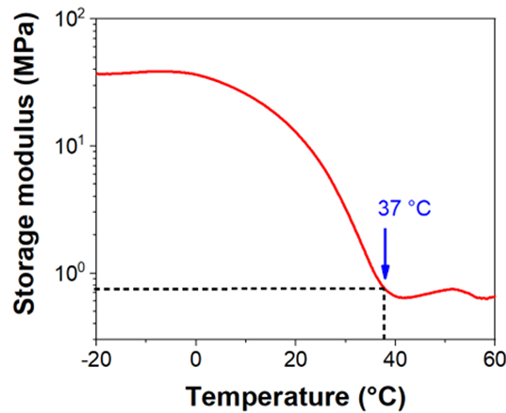


Figure S3. The dynamic mechanical analysis curves of softening polymer substrate.


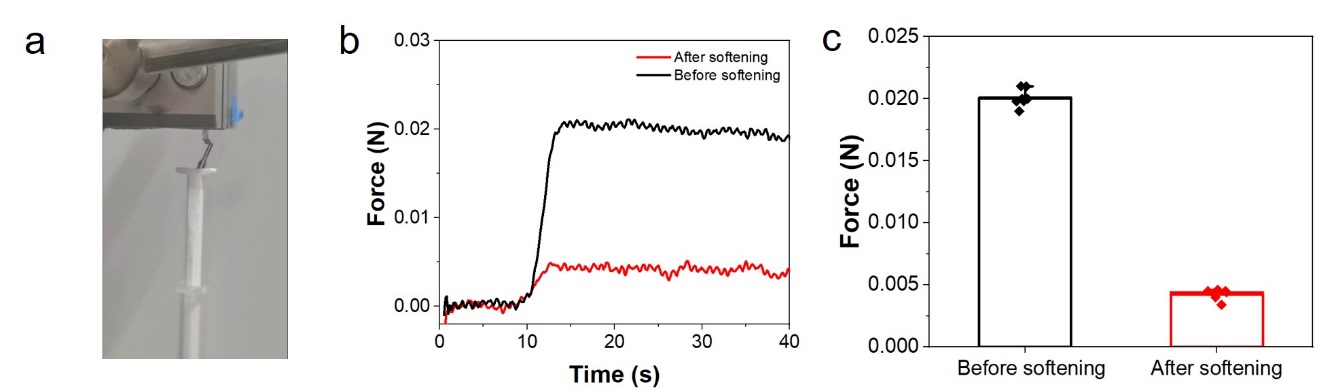


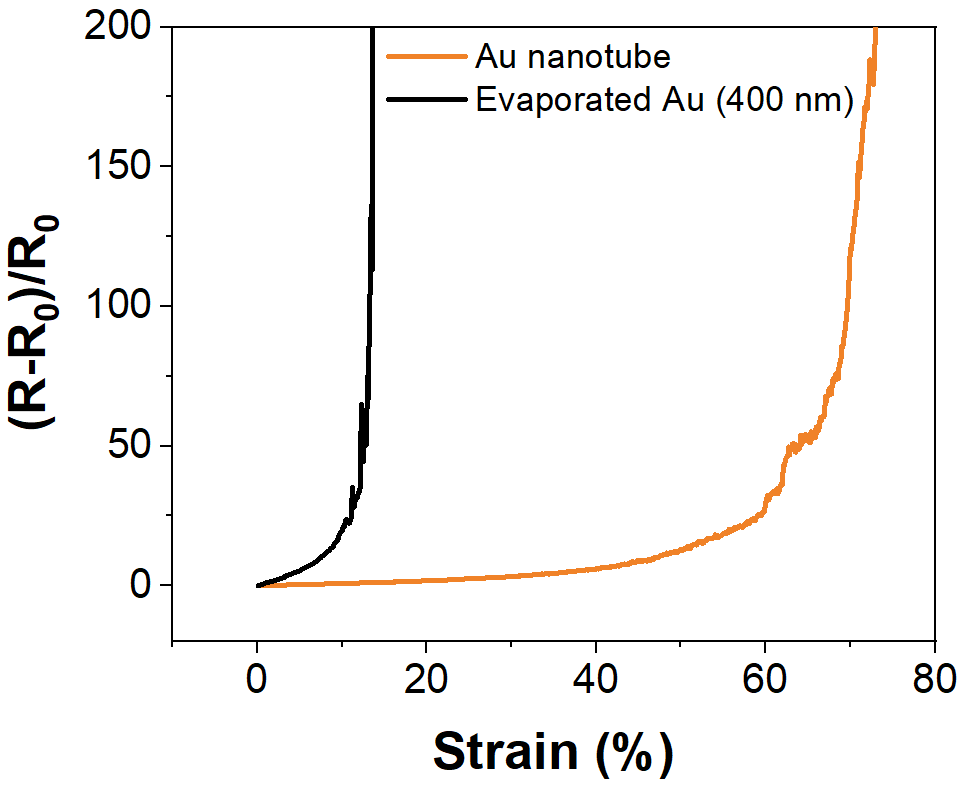
Figure S4. The stress of our BIS-PNE before and after softening. (a) Measure method. (b, c) Force-time curves (b) and force (c) before and after softening. Data in (c) are presented as mean ± s.d. from 7 samples.

Figure S5. The relative resistance changes of the Au nanotube layer and evaporated Au layer composite with polymer substrate.


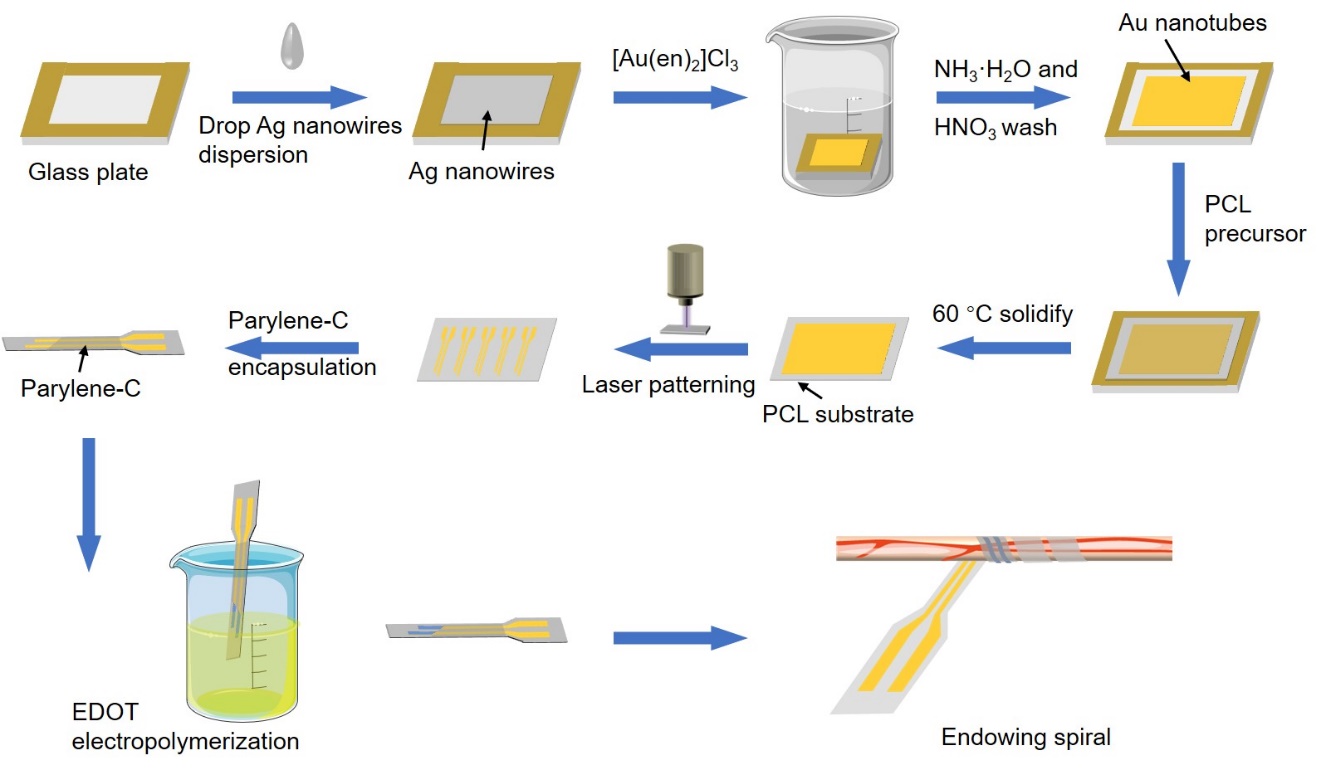


Figure S6. Schematic illustrations of the detailed fabrication process of the body temperature softening peripheral nerve electrode.


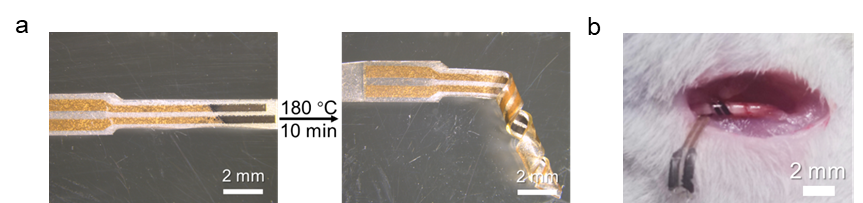
 Figure S7. (a) The stereomicroscope images of the planar BIS-PNE transfer helical BIS-PNE under 180 °C for 10 min. (b) The optical image of our BIS-PNE twining sciatic nerve of the rat.


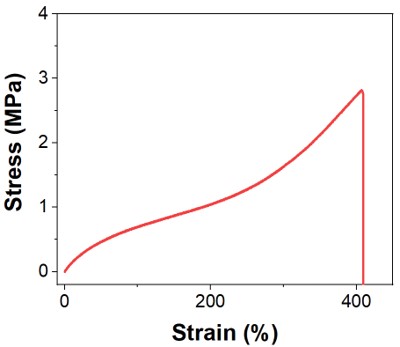


Figure S8. The stress-strain curve of the polymer substrate at 37 °C.


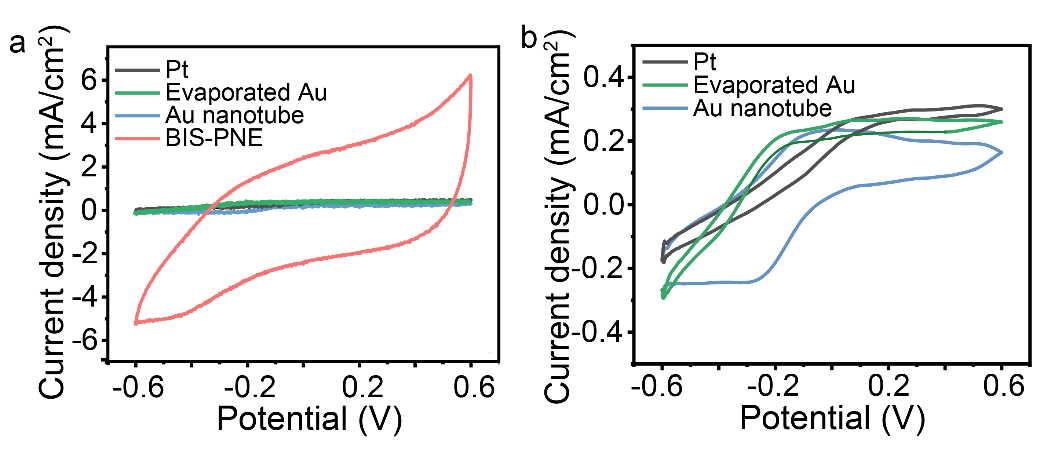


Figure S9. The CV curves of our BIS-PNE, Pt, evaporated Au, and Au nanotube electrodes.


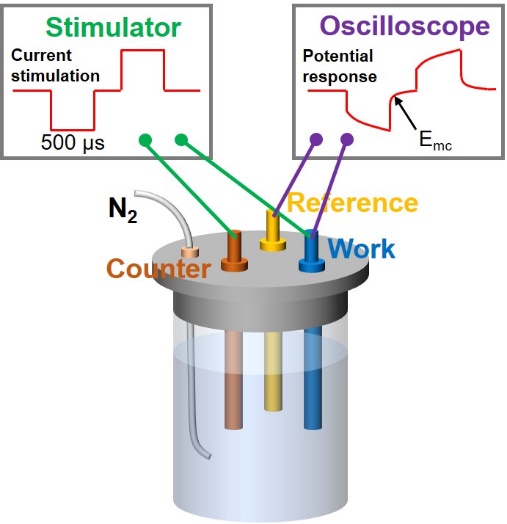


Figure S10. Schematic diagram of the cathode charge injection capacity test device.


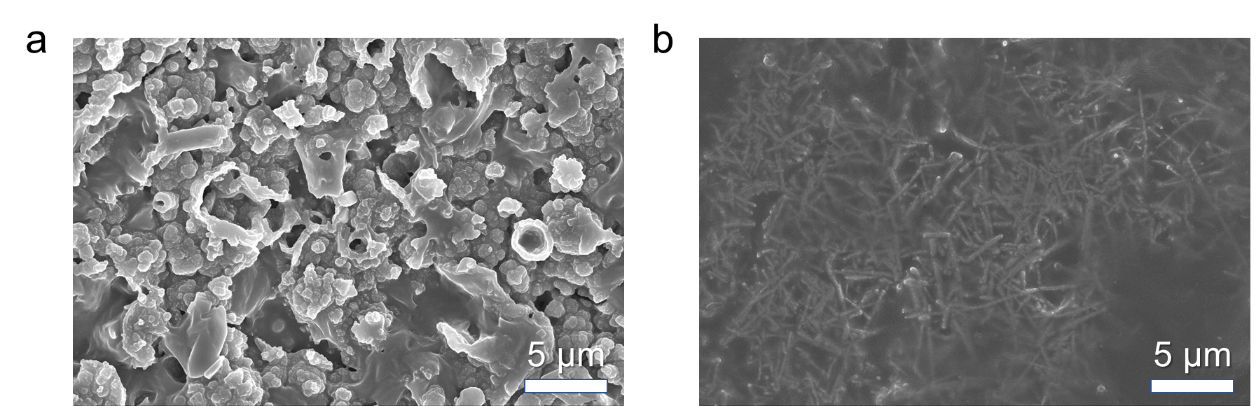


Figure S11. SEM images of BIS-PNE (a) Au nanotube electrode (b).


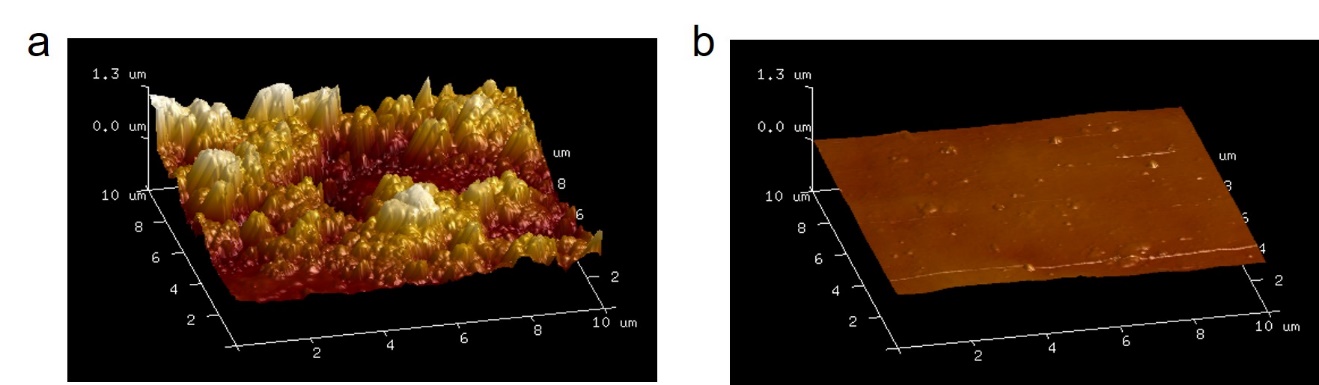


Figure S12. AFM 3D height images of BIS-PNE (a) and Au nanotube electrode (b).


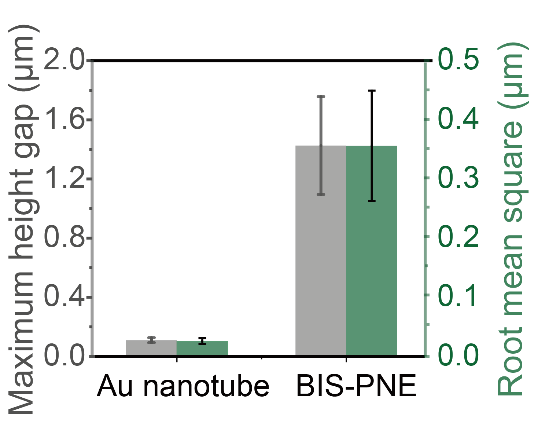


Figure S13. Maximum height difference and root mean square roughness of Au nanotube electrode and our BIS-PNE. Data are presented as mean ± s.d. from 7 samples.


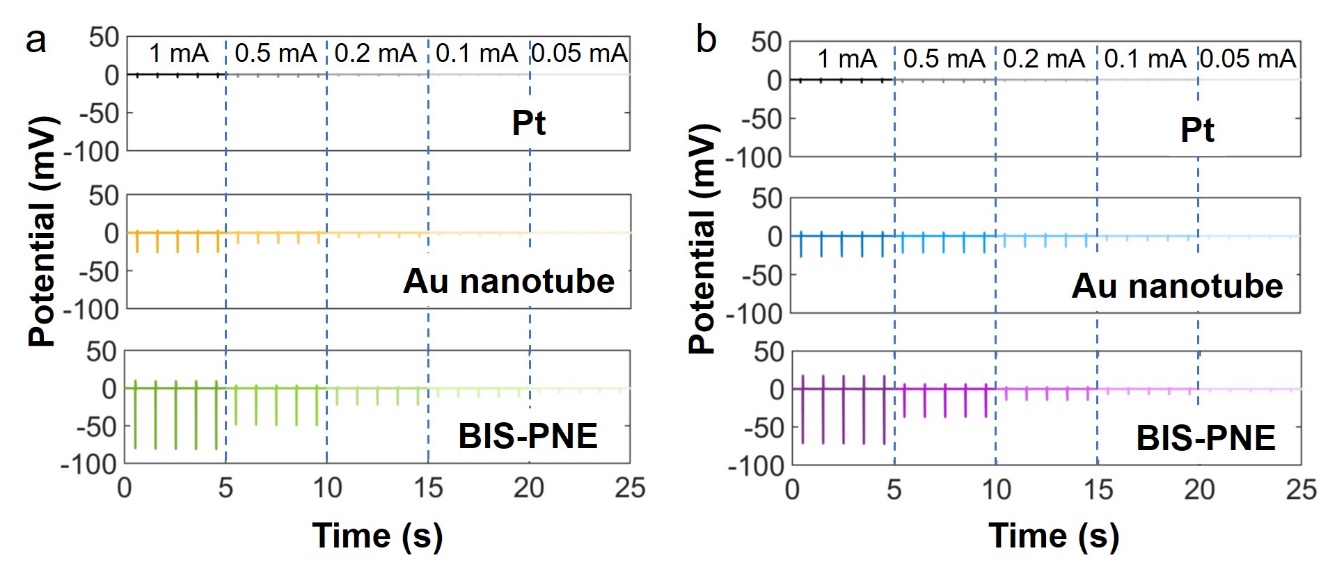


Figure S14. (a) Sciatic nerve action potentials recorded with Pt, Au nanotube electrodes, and BIS-PNE, evoked by Pt electrode stimulation at 0.05~1 mA. (b) Sciatic nerve action potentials recorded with Pt electrode, evoked by Pt, Au nanotube, and BIS-PNE at 0.05~1 mA.


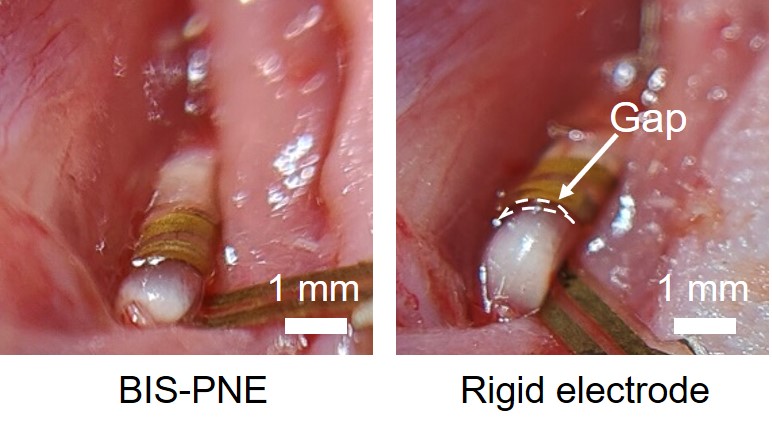


Figure S15. BIS-PNE conformally wraps the tissue with the robust interface (left). The rigid electrode does not wrap snugly around the tissue and leaves gaps (right).


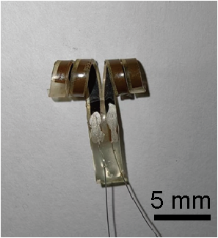


Figure S16. The optical image of the BIS-PNE used for canine experiments.


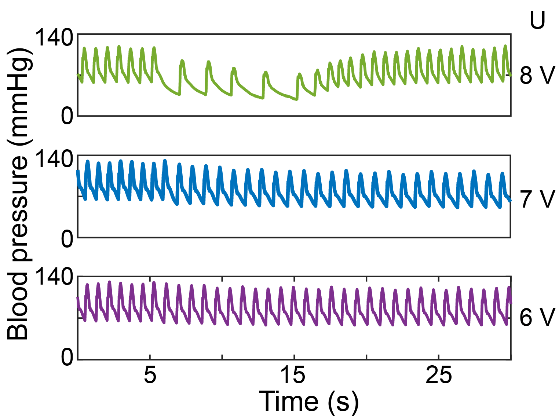


Figure S17. The real-time monitoring of blood pressure when stimulated with Pt electrode in various voltages.


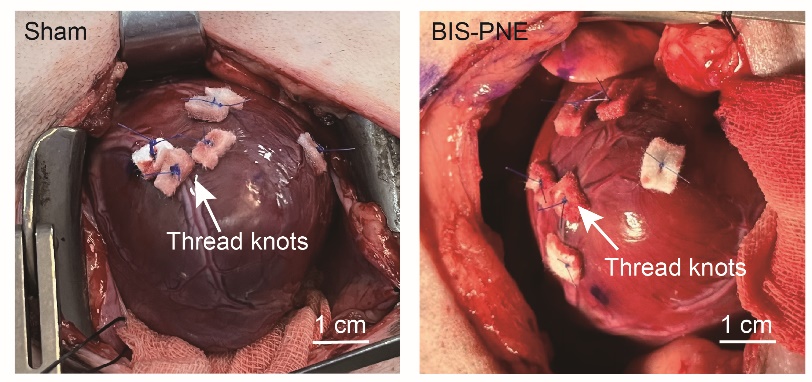


Figure S18. Optic images of anterior descending artery ligation.


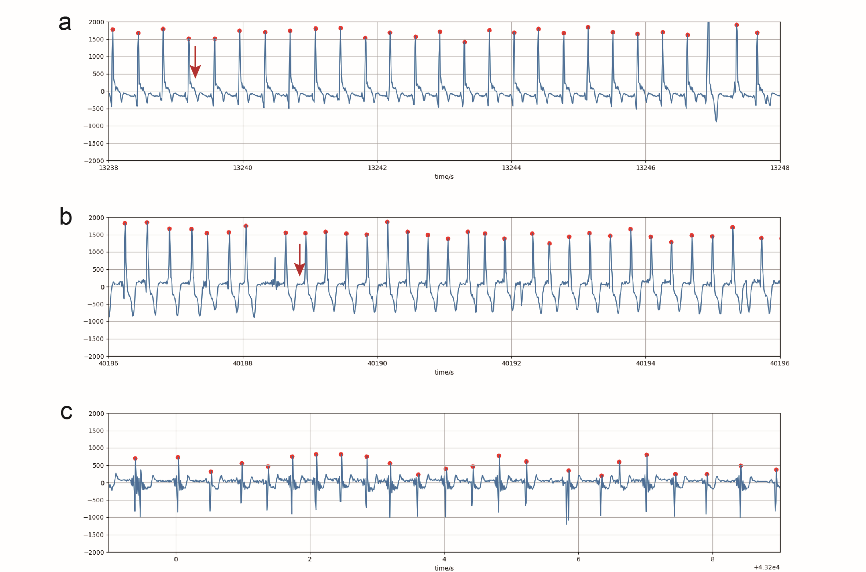


Figure S19. The typical ECG of a dog with MI (a). The red arrow presents the S-T segment elevation. (b) The ECG of the dog in the sham group during VT. The red arrow presents the absence of the P pulse. (c) The ECG of the dog in the BIS-PNE group.


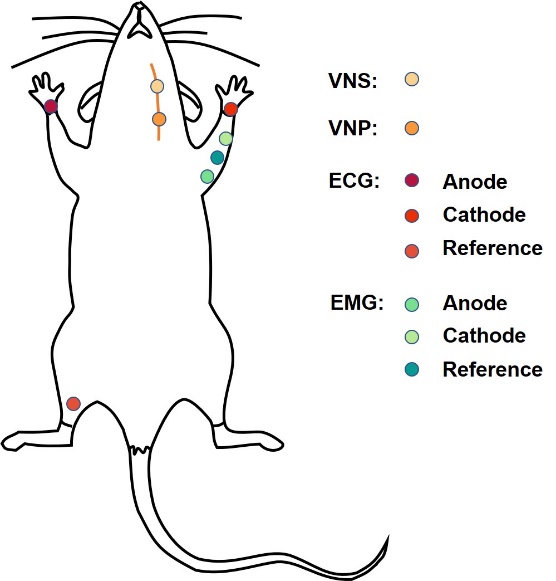


Figure S20. Schematic of vagus nerve stimulation (VNS) and vagus nerve potential (VNP), electrocardiogram ECG, and electromyogram EMG recording in the epileptic rat model.


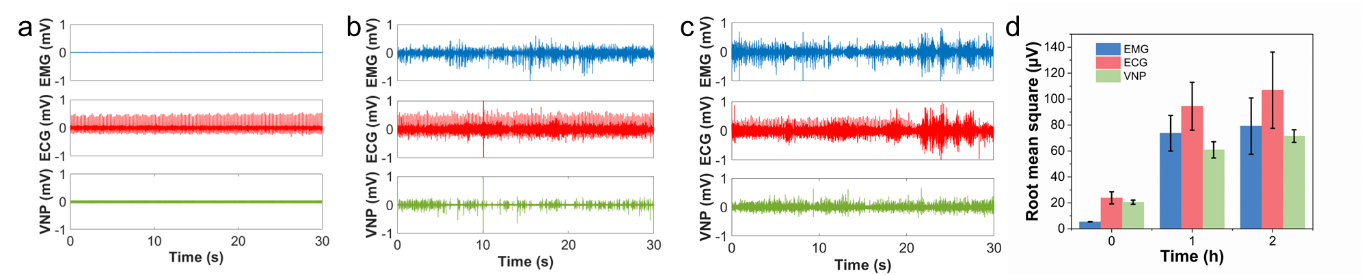


Figure S21. EMG, ECG and VNP at 0 h (a), 1 h (b) and 2 h (c) after pilocarpine injection in the control group. (d) The root mean square of EMG, ECG, and VNP of the control group. Data in d are presented as mean ± s.d. from 5 samples.


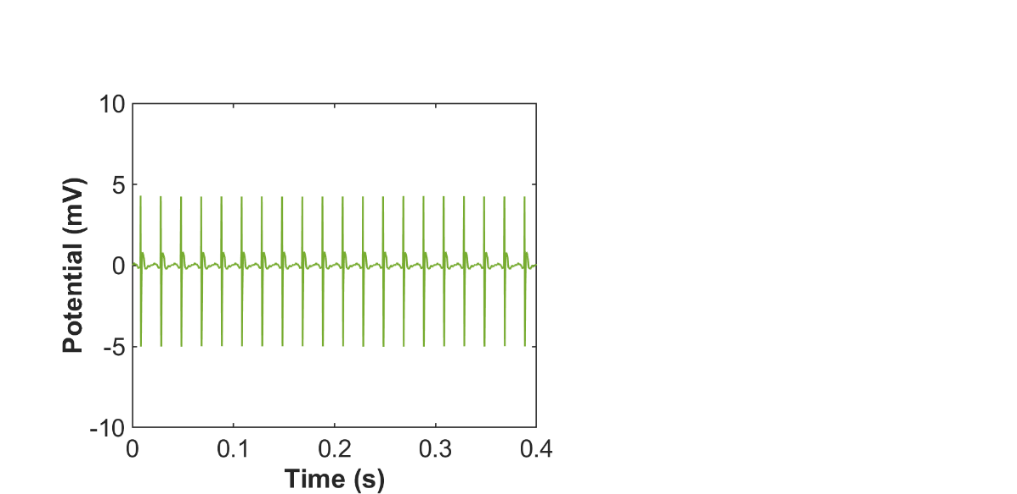


Figure S22. VNP recordings under VNS.


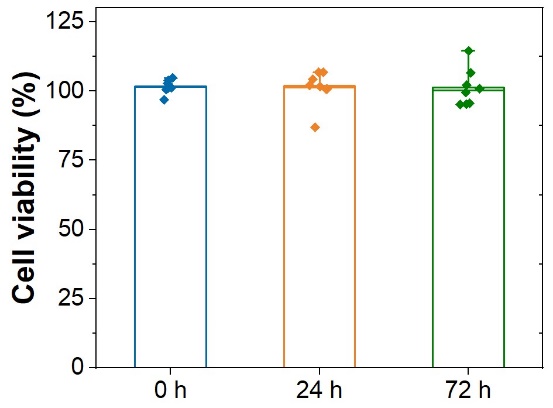


Figure S23. CCK-8 assay quantification of cell viability at 0, 24, and 72 h. Data are presented as mean ± s.d. from 8 samples.


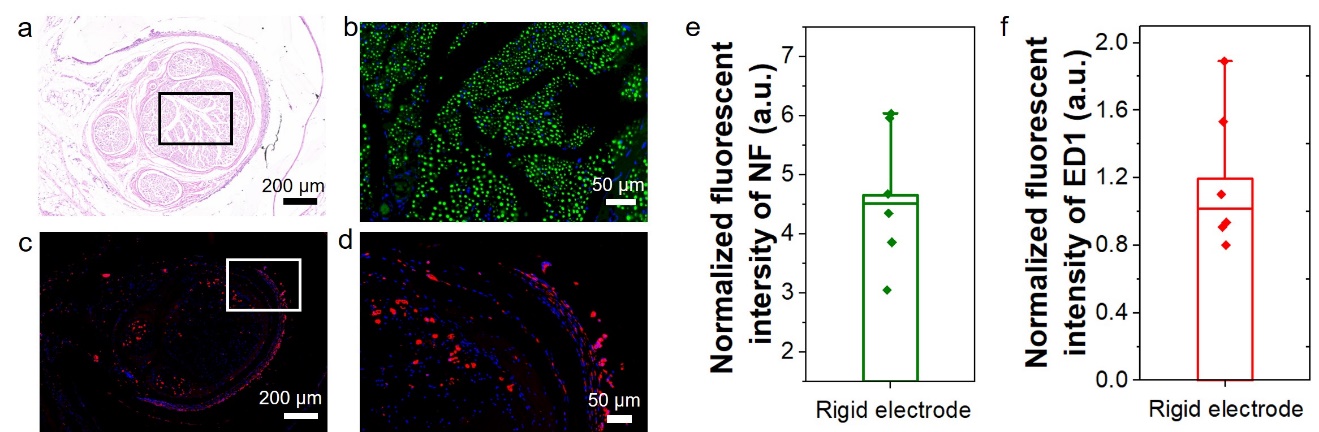


Figure S24. Cross-sectional slices of the sciatic nerve implanted with the rigid electrode. H&E staining (a), fluorescent images immunochemically labeled by the biomarker neurofilament (NF) and 4’,6-diamidino-2-phenylindole (DAPI) (b), fluorescent images immunochemically labeled by the biomarker ED1 and DAPI (c), and magnified images (d). (e, f) Histogram showing the normalized fluorescence intensity of NF (e) and ED1 (f) for rigid electrode groups. Data in (e) and (f) are presented as mean ± s.d. from 6 samples.


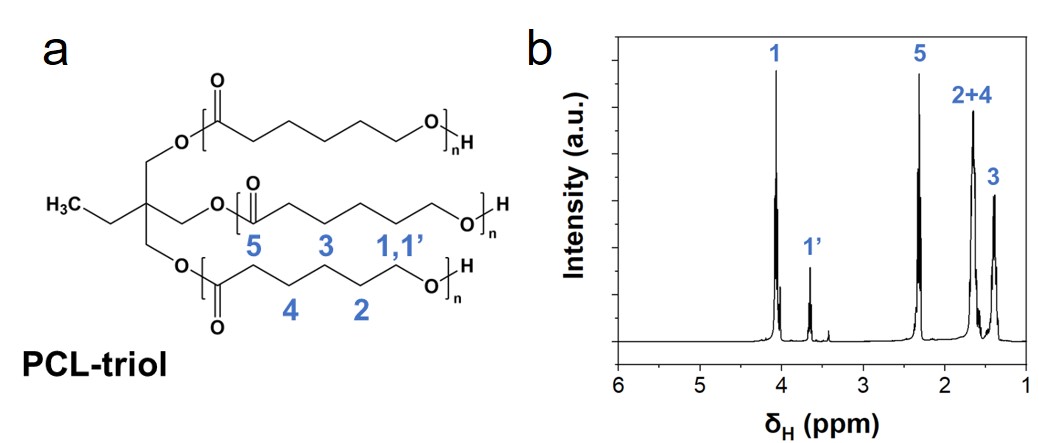


Figure S25. PCL-triol molecular structure (a) and corresponding H1 nuclear magnetic resonance (NMR) spectra (b).
